# Differential gene expression of ABCG2, SLC22A12, IL-1β, and ALPK1 in peripheral blood leukocytes of primary gout patients with hyperuricemia and their comorbidities: a case–control study

**DOI:** 10.1186/s40001-022-00684-1

**Published:** 2022-05-03

**Authors:** Paniagua-Díaz Natsuko, Sanchez-Chapul Laura, Clavijo-Cornejo Denise, Ventura-Ríos Lucio, Aguilar-Salinas Carlos, Sanchez-Muñoz Fausto, López-Macay Ambar

**Affiliations:** 1 Laboratorio de Enfermedades Neuromusculares, Instituto Nacional de Rehabilitación, Guillermo Ibarra Ibarra. Calzada Mexico-Xochimilco 289, Colonia Arenal de Guadalupe, División Neurociencias, CP 143898 Ciudad de México, México; 2 Division of Musculoskeletal and Rheumatic Diseases, Instituto Nacional de Rehabilitación, Mexico City, Mexico., Instituto Nacional de Rehabilitación – “Luis Guillermo Ibarra Ibarra”. Tlalpan, Ciudad de México, México; 3 Laboratorio de Ultrasonido Musculoesquelético Articular, Instituto Nacional de Rehabilitación “Luis Guillermo Ibarra Ibarra”, Tlalpan, Ciudad de México, México; 4 Unidad de investigación de enfermedades metabólicas, Instituto Nacional de Ciencias Médicas Y Nutrición Salvador Zubirán. Tlalpan, Ciudad de Mexico, México; 5 Department of immunology, Instituto Nacional de Cardiología Ignacio Chávez, Mexico City, 14080 Tlalpan, Ciduad de México, México

**Keywords:** Asymptomatic gout, ALPK1, ABCG2, SLC22A12, Metabolic syndrome

## Abstract

**Background:**

The ABCG2, SLC22A12, and ALPK1 genes have been strongly associated with dysfunction of urate metabolism in patients with gout, but it is unknown how these transporters are expressed in patients with acute or chronic gout. Our objectives were to: (a) analyze the gene expression of urate transporters and of inflammation genes in peripheral blood from gout patients and controls; (b) determine whether the metabolic profile of gout patients can influence the gene expression profile and the expression of urate transporters, ABCG2 and SLC22A12, and inflammation molecules, ALPK1 and IL-1β, in peripheral blood leukocytes from gout patients; (c) compare them with their metabolic profile and the gene expression of people without gout and without hyperuricemia.

**Methods:**

A total of 36 chronic and acute patients and 52 controls were recruited, and ABCG2, SLC22A12, IL-1β, and ALPK1 gene expression was evaluated by quantitative real-time PCR. Correlations of gene expression with clinical and laboratory parameters of patients were also analyzed.

**Results:**

IL-1β was significantly increased in peripheral blood mononuclear cells (PBMCs) of patients compared with their polymorphonuclear leukocytes white blood cells (PMNLs, *p* < 0.05). A significant increase in ABCG2 and IL-1β was found in PMNLs from patients compared to controls (*p* < 0.05). Correlations of gene expression in patients were found with levels of serum uric acid (sUA), serum creatinine, C-reactive protein (CRP), triglycerides, body mass index (BMI), kidney disease, hypertension, and metabolic syndrome.

**Conclusions:**

Our data suggest that leukocytes of patients respond to the presence of hyperuricemia and comorbidities, expressing ABCG2 and IL-1β genes differentially compared to normouricemic and nondisease states. Hyperuricemia, dyslipidemia, and obesity probably stimulate the differential gene expression of peripheral blood leukocytes (neutrophils and monocytes), even in an asymptomatic state.

## Background

Gout is a multifactorial disease characterized mainly by acute joint pain due to the immune response activated by the precipitation of monosodium urate crystals (MSU) in the joints. This disease is currently associated with components of the metabolic syndrome, such as obesity, hypertension, and cardiovascular diseases [1, 2]. Worldwide, gout is a frequent disease, reaching a prevalence of over 1% in developed countries and up to 3.9% in the USA [3]. In Mexico, the reported prevalence is 0.35%, although it is suggested that it may reach 3% [4, 5]. The inflammatory mechanisms of gout are known from in vitro studies and in animal models [6], however scarce studies exist on inflammatory events outside the joint that could serve as indicators of damage or of disease evolution. The intestine, blood, and saliva of patients can be exposed to high concentrations of soluble uric acid, even in an asymptomatic state. However, little is known about the cellular and molecular events involved in these conditions [7–9].

In recent years, genes related to the reabsorption and elimination of uric acid in the kidney and its association with gout have been studied [10–13]. Currently, the function of each of these genes and their possible role in the gout etiology have begun to be studied in vitro or animal models [14–17]. Human genomic studies have linked various urate transporter genes such as ABCG2, SLC22A12 (urate transporter 1: URAT1 protein), SLC2A9 (GLUT9), OAT4, among others, as possible risk factors [12, 18, 19]. Although low basal expression of urate transporters such as ABCG2 and SLC22A12 has been reported in peripheral blood of healthy people, we do not know if their leucocyte expression is increased in patients with gout and hyperuricemia and this may show some altered local cellular process; likewise, it is unknown whether these changes in expression are dependent on an inflammatory process or only depend on a hyperuricemic state or both.

The cytokine IL-1β participation in the response to MSUs in gout attack has long been known, but little is known about its effect on ABCG2 and its interaction with other urate transporters. Studies in human intestinal ABCG2-knockdown cells stimulated with MSU showed increases in IL-1β and IL8; therefore, this and other studies suggest a relationship between transporters and inflammatory cytokines [14, 20]. ALPK1 kinase has been associated with the regulation of the expression of URAT1 and testosterone in the kidney in murine models [21–24], hence, there seems to be an interaction between the expression of inflammatory molecules and the activation of transporters at least in the kidney and intestine [25]. Monocytes and neutrophils are important cells in the inflammatory microenvironment of gout and are found in a large percentage in peripheral blood. Both cell groups can induce their proinflammatory activation depending on different proinflammatory stimuli; however, monocytes have the main function of differentiating into macrophages or dendritic cells and amplifying the immune response, whereas for the latter, their main function is to directly eliminate microorganisms [26–28]. We hypothesized that there are differences in the gene expression of transporters and proinflammatory molecules in leukocytes (PBMCs or PMNLs) between gout patients and controls and that these differences are related to hyperuricemia.

## Methods

### Study design

This is an a cross-sectional and observational study of case and controls where 36 patients and 52 controls were included. This study was conducted at Rheumatology department, Laboratorio Musculoesquelético y Articular, and Laboratorio de Enfermedades Neruomusculares of National Institute of Rehabilitation “Luis Guillermo Ibarra Ibarra” in Mexico city. The study was approved by the local ethical committee with number 51/14 and conducted according to the Declaration of Helsinki. All participants gave written informed consent before study inclusion.

### Inclusion and exclusion criteria

All patients have gout according to ACR/EULAR 2015 criteria: (1) the presence of MSU in the synovial fluid using a polarized microscope or (2) at least six of 12 clinical criteria being met [29, 30]. The exclusion criteria were (a) autoimmune diseases such as rheumatoid arthritis or systemic lupus erythematosus; (b) bone dysplasias; (c) diabetes mellitus, and (d) kidney stones. Hospital control group was conformed by healthy people (they did not have hyperuricemia, gout, joint diseases, heart disease or kidney stones). Informed consent was obtained from all individual participants included in the study.

### Patient evaluation

Patient demographics, clinical and biochemical were obtained for all participants. The clinical examination included height, weight, body mass index (BMI) in kg/cm^2^. All comorbidities were recorded as well as smoking, alcohol consumption, disease duration and uric acid level. All patients underwent a clinical evaluation to establish or rule out the diagnosis of MS at the time of the study, according to the criteria of the Adult Treatment Panel III report (ATP III) [29].

Determination of clinical parameters/biochemical data was performed with a UniCel DxC 600 device, Synchron Clinical System. A peripheral blood sample was taken from fasting participants for metabolic parameters including serum uric acid (sUA), glucose (GLC), total cholesterol (TC), triglycerides (TG), high lipoprotein density (HDL), low density lipoprotein (LDL), creatinine (Cr) and C-reactive protein (CRP).

### Isolation of cells and RNA extraction

The PBMCs and PMNLs were isolated using gradient centrifugation (1.077 g/ml) with Polymorphprep. Total RNA of human PBMCs and PMNLs was extracted using NucleoSpin Kit (MACHEREY–NAGEL, Düren, Germany). RNA obtained was preserved at −20 °C until RTqPCR was performed.

### Analysis of gene expression by RT PCR

Total RNA, from mononuclear and polymorphonuclear cells of peripheral blood, was amplified by RTqPCR using GoTaq System real kit (Promega, Madison, WI, USA) and the Qiagen Rotor Gene Q kit (Qiagen, Venlo, The Netherlands). The amplification conditions were as follows: 37 °C reverse transcription for 15 min, activation of Taq Polymerase at 95 °C for 10 min, 45 cycles of denaturation at 95 °C for 10 s, alignment at 62 °C for 10 s, and elongation at 72 °C for 10 s. The primers are presented in Table 1.

**Table 1:** Primers sequence

Gene	Direcction	Secuence (5′–3′)
GAPDH	Forward	GTATGACAACGAATTTGGCTACAG
	Reverse	GTCTCTCTCTTCCTCTTGTGCTCT
SLC22A12	Forward	TGGTGCTAACCTGGAGCTACC
	Reverse	TGTTCATCATGACGCCTGC
ABCG2	Forward	TCTCTTCTTCCTGACCTGCTG
	Reverse	AAACCACACTCTGACCTGCTG
SLC2A9	Forward	CTGTCTGGGTCGGACACTCG
	Reverse	CTTGCGTTCCTTCCGGGTTG
SLC22A3	Forward	ATCCCGGGCACACATTCCAT
	Reverse	GTTGGAGCAGCCCTGGAGAA
ALPK1	Forward	CGGCACAGTCTGGTCCTTTG
	Reverse	CAGAAGCAGCGGTCTCCTGA
TLR4	Forward	TGAGCAGTCGTGCTGGTCTC
	Reverse	CAGGGCTTTTCTGAGTCGTC
IL1β	Forward	GGCCATCAGCTTCAAAGAAC
	Reverse	GAGCTCGCCAGTGAAATGAT

The ΔCt method of Livak and Schmittgen was used to compare mRNA expression of the target genes in patients versus controls. The ΔΔCt method was used to compare mRNA expression between target genes and PMNL versus PBMC. GAPDH gene is an internal control to normalize the expression of each gen.

### Statistical analysis

Descriptive statistics were applied for analysis of demographics, clinical and biochemical data. We used Shapiro–Wilk to ensure the normality of the data and followed by Student’s t test, Mann–Whitney *U* test or one-way analysis of variance (ANOVA) and Bonferroni–Games–Howell post hoc test. The results were considered significant when *p* < 0.05, *p* < 0.01 or *p* < 0.001. All tests were performed with SPSS 22 software (SPSS, Inc., Chicago, IL, USA). The calculation of the statistical power *p* = (1−β) in the analysis of gene expression was carried out using the GPower 3.1.9.2 program.

The correlations between the clinical data, clinical history and expression levels of the evaluated population were made with Spearman's rho test or Pearson’s test. The values are significant at *p* < 0.05 or *p* < 0.01.

## Results

### Clinical characteristics and biochemical parameters of patients and controls

A total of 36 patients and 52 controls participated in the study. Clinical characteristics and biochemical parameters are presented in Table 2. In general, patients with gout presented a higher percentage of obesity (35.3%), hyperuricemia (7.88 ± 0.34 mg/dl), high TGs (244.45 ± 28.92 mg/dl), very low HDL (38.15 ± 1.69 mg/dl) and high creatinine above the reference values (1.21 ± 0.18) than controls. These values were significantly higher than those in the controls, as shown in Table 3.

**Table 2:** Characteristics of the overall study population

		**Controls** ***N* = 52**		**Patients** ***N* = 36**
	n		n	
Age, years (Media ± SD)	49	33.71 ± 1.55	36	45.56 ± 2.21 *
Gender (%) Male Female	43 9	82.69 17.30	35 1	97.22 2.77
Gout onset time/Age (years)	NA	NA	29	8.6, 37.02
gout flare (%) Chronic Gout (%)	NA NA	NA NA	7 29	19.44 80.56
Normal weight (%)	30	56.6	8	23.5*
Overweight (%)	19	35.8	14	41.2
Obesity (%)	4	7.4	12	35.3*
Hypertensión (%)	1	2.17	8	22.22*
Metabolic syndrome (%)	0	0	11	29.7*
Presence of tophi (%)	NA	NA	10	27.8
Renal disease (%)	NA	NA	4	10.8
Diabetes mellitus (%)	0	0	0	0
Alcohol consumption(%) Yes No				
	17	33.3	14	42.4
	34	66.07	19	57.6
Smoking (%) Smoker Non smoker Ex smoker				
	6	11.5	10	27*
	40	76.9	18	48.6*
	6	11.5	9	24.3
Gout background in the family (%) Yes No				
	4	8.2	16	45.7*
	45	91.8	19	54.3*
Medications				
allopurinol	NA	NA	7	19.44
aspirin	NA	NA	2	5.55
colchicine	NA	NA	3	8.33
hydrochlorothiazide	NA	NA	2	5.55
bezafibrate	NA	NA	1	2.77
Losartan	NA	NA	1	2.77
prednisone	NA	NA	1	2.77
indomethacin	NA	NA	1	2.77
metoprolol	NA	NA	1	2.77

The p values were estimated by one-way analysis of variance (ANOVA) and posterior comparison was estimated by Bonferroni correction (significance for *p* < 0.05). NA = does not apply

**Table 3:** Demographic datas and clinical characteristics of subjects in the study

		Controls *N* = 52		Patients *N* = 36
	**n**		**n**	
SERUM				
Uric Acid (mg/dL)	52	5.57 ± 0.17	36	7.88 ± 0.34*
Glucose (mg/dL)	52	86.96 ± 1.24	36	88.76 ± 2.99
Cholesterol (mg/dL)	52	193.04 ± 5.29	35	188.27 ± 7.08
Triglycerides (mg/dL)	52	141.73 ± 13.89	35	244.45 ± 28.92*
Creatinine (mg/DL)	52	0.95 ± 0.08	35	1.21 ± 0.18*
HDL (mg/dL)	50	50.86 ± 1.96	33	38.15 ± 1.69*
LDL (mg/dL)	50	115.50 ± 4.16	33	101.35 ± 33.00**
CRP (mg/L)	23	5.00 ± 2.7	26	5.98 ± 4.6
ORINE				
Uric Acid (mg/24 h)	NA	NA	7	881.61 ± 621
Creatinine Clearance (cc/min)	NA	NA	4	123.95 ± 50

NA = does not apply.The values represent the mean ± SD. The p-values were estimated with Student’s T test and U Mann Whitney test. ** *p* < 0.001, * *p* < 0.01

A total of 15 patients had a prescription variable of drugs, such as monoallopurinol prescription (300 mg/day), indomethacin (50 mg/day), losartan (50 mg/day), metoprolol (100 mg/day), prednisone (50 mg/day), bezafibrate (400 mg/day) colchicine (1 mg/day) and aspirin (> 325 mg/day).

### Analysis of gene expression in PBMC and PMNL of patients and controls

**Fig. 1:**
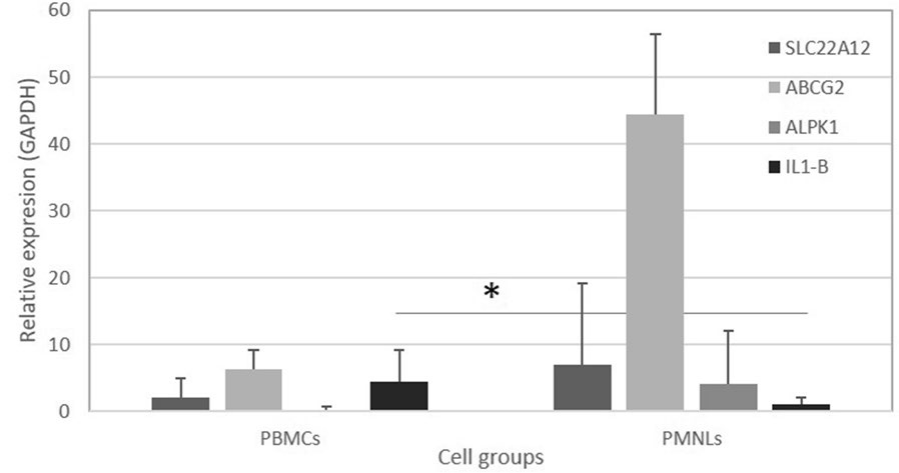
Comparison of the gene expression of ABCG2, SLC22A12, ALPK1 and IL-1β between PBMCs and PMNLs from patients. The figure shows the average and standard error (SE). * represents significant differences (*p* < 0.01)

We compared relative expression of PBMC and PMNL, as shown in Fig. 1. IL-1β expression was significantly increased, *p* < 0.01 in PBMCs compared to PMNLs in patients; therefore, the other genes were expressed in a similar way between different cell groups.

**Fig. 2:**
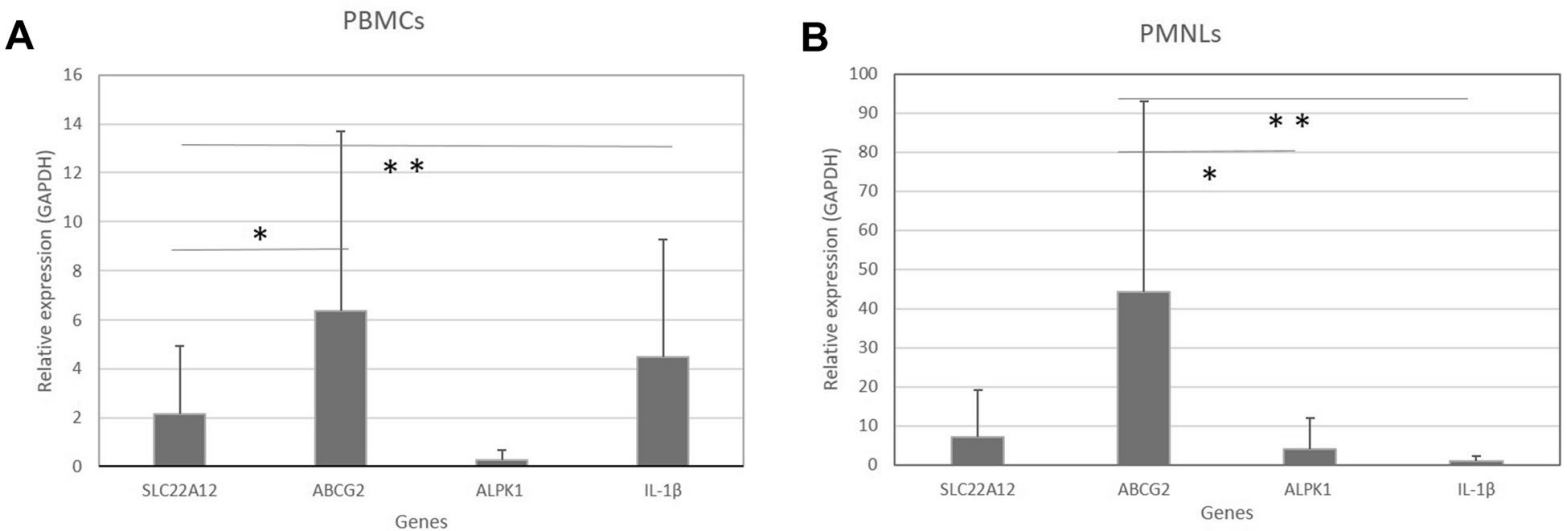
Comparison of expression between genes in PBMCs and PMNLs from gout patients. The relative expression of all genes evaluated in PBMCs (**A**) and PMNLs (**B**) is shown. * represents significant differences, ** (*p* < 0.01), *(*p* < 0.05). The graph shows the average and SE

Our next analysis was the gene expression comparison of the 4 genes between both cell groups in patient samples. In PBMCs, ABCG2 was expressed more than the other genes, but was only significantly higher with respect to the URAT1 gene (*p* < 0.05), while IL-1β was significantly higher than SLC22A12 (*p* < 0.05), as shown in Fig. 2. In PMNLs, ABCG2 was expressed more than the other genes, but it was only significant with respect to ALPK1 (*p* < 0.05) and IL-1β (*p* < 0.01).

**Fig. 3:**
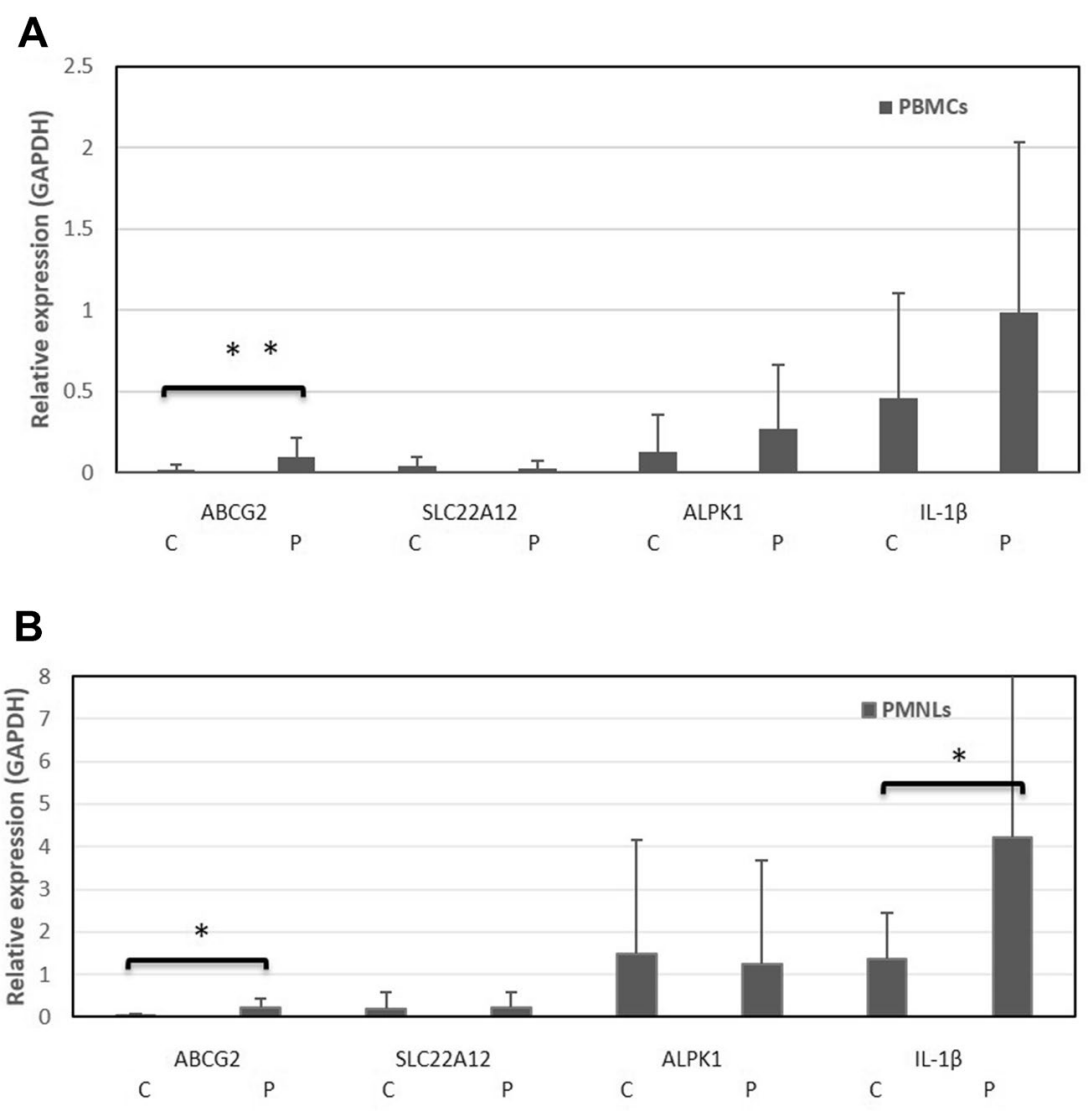
Comparison of the gene expression of patients vs controls in PBMCs and PMNLs. C = controls, *p* = patients. Analysis of relative expression in PBMCs (**A**) and PMNLs (**B**) of patients and controls. *represents significant differences (*p* < 0.05) or ** (*p* < 0.01). The graph shows the average and SE

Finally, gene expression was analyzed to determine whether transporter genes increase their expression in the blood due to frequent exposure to high concentrations of uric acid and due to the presence of dyslipidemia and metabolic syndrome. We analyzed the gene expression of ABCG2, ALPK1, SLC22A12 and IL-1β in PMNLs and PBMCs to compare the results between patients and controls, as shown in Fig. 1. ABCG2 expression in PMNLs was significantly higher (*p* < 0.05) in patients (*n* = 18) than in controls (*n* = 18), with *p* = 0.8875. In PBMCs, ABCG2 had a significantly higher expression (*p* < 0.05) in patients (*n* = 15) than in controls (*n* = 13) (Fig. 2). IL-1β expression in PMNLs was significantly higher (*p* < 0.05) in patients (*n* = 13) than in controls (*n* = 14). We did not find differences in the SLC22A12 expression between PBMCs and PMNLs, as seen in Fig. 3.

A correlation analysis of gene expression was performed with the biochemical parameters and the clinical history of the patients and the total population (patients and controls). The results are shown in Table 4.

**Table 4:** Significant correlations between gene expression and clinical data

	Patients			Total population		
	Correlation coeficient	Significant level (2 tailed)	n	Correlation coeficient	Significant level (2 tailed)	n
ABCG2						
Creatinin (PBMCs)	0.915 ^#^	*p* < 0.001	18	0.875 ^#^	*p* < 0.001	34
Uric Acid (PMNLs)	NS	NS	18	0.421 ^§^	*p* < 0.05	35
SLC22A12						
MS (PBMCs)	0.473 ^§^	*p* < 0.05	20	NS	NS	34
UA (PBMCs)	0.449 ^§^	*p* < 0.05	21	NS	NS	35
TAG (PBMCs)	0.466 ^#^	*p* < 0.05	21	NS	NS	34
TAG (PMNLs)	0.550^§^	*p* < 0.05	15	NS	NS	34
RD (PMNLs)	0.525 ^#^	*p* < 0.05	16	0.663 ^§^	*p* < 0.001	15
Hypertension (PMNLs)	0.511 ^#^	*p* < 0.05	16	NS	NS	36
ALPK1						
Creatinin (PBMCs)	0.654 ^§^	*p* < 0.001	16	NS	NS	34
BMI (PBMCs)	NS	NS	18	0.443 ^§^	*p* < 0.001	39
Uric Acid (PBMCs)	0.574 ^§^	*p* < 0.05	16	NS	NS	37
CRP (PMNLs)	0.620 ^§^	*p* < 0.05	12	NS	NS	30
IL-1β						
Hypertension (PMNLs)	0.579 ^§^	*p* < 0.05	15	NS	NS	35

^§^Correlation coeficient Spearman^, #^Correlation coeficient Pearson. *NS* non significant

## Discussion

All the volunteers in this study were Mexican and lived in Mexico City. Mexico has approximately 70% of its adult population between 30 and 60 years old who are overweight [30, 31]). The clinical data analysis of the patients showed that the subjects did not have adequate treatment or follow-up of their comorbidities associated with gout, since regardless of the age or stage of the disease, acute gout was (15.55%) or asymptomatic (84.44%), and we found metabolic alterations in most of them [1, 12, 32]. A total of 67.56% of the patients presented hyperuricemia, 75% hypertriglyceridemia, 67.64% low HDLs, 19.44% high creatinine, and a high percentage of obesity (35.3%) and overweight (41.2%), and 10.8% of them had kidney disease previously diagnosed. It is important to mention that only 18.91% had hyperglycemia and that none of them had a diagnosis of diabetes. (Therefore, this cohort of patients with asymptomatic or acute gout had poor monitoring of their comorbidities, which puts them at risk of new episodes of gout and of developing cardiovascular and kidney diseases. The percentage of people with a family history of gout in our study was 45.7%).

The profile of the patients in this study showed that despite differences in time with the disease, type of treatment, and symptomatology, ABCG2 gene expression was higher in normouricemic controls. ABCG2 was the gene that yielded the most important findings because it showed greater expression than the others in PMNLs and PBMCs and with respect to the controls in both cell groups. We interpret this as a response of different cell types to the presence of uric acid at high concentrations in serum. It is known that salivary and brain cells respond by overexpressing ABCG2 to increasing uric acid levels in their environment so that different cells can induce the expression of the gene under similar conditions [33, 34]. Chen et al. reported that exposure to sUA increased the expression of ABCG2 in intestinal cells, which activated the inflammasome and the PI3K/Akt pathway, thus, there is a possibility that soluble uric acid can activate the expression of ABCG2 in peripheral blood leukocytes [14]. Unlike controls, in patients, there is a high probability of the presence of inflammation caused by hyperuricemia or its comorbidities, which may favor the expression of ABCG2. Proinflammatory factors, such as TNF-α, IL-1β, and IL-6 have been reported to increase ABCG2 expression in various in vitro models [8–10]. It is possible that ABCG2 increases its expression in blood and tissues in a state of hyperuricemia, trying to eliminate sUA in different cell types. In people with mutations that decrease the elimination of urates, hyperuricemia could be latent. Ultimately, high concentrations of uric acid activate the innate immune response by overexpressing IL-1β in neutrophils and monocytes, which favors an acute episode of gout if urate homeostasis is not regulated.

Our PBMCs data suggest that the increase in the expression of ABCG2 in blood from patients could be related to the presence of renal dysregulation. We found a positive correlation of the expression of ABCG2 with the creatinine levels of patients, and this correlation was maintained when analyzed with the total study population. The mean levels of serum creatinine were found to be high in patients, Table 3; from the demographic data of the patients, we know that the history of kidney disease ranged from 10 to 16%, as shown in Table 2. These data in PBMCs suggest that the increased ABCG2 expression in blood could be related to the presence of renal dysregulation. Understanding the role of ABCG2 in different organs of patients with gout will allow knowing the key points of its dysregulation. A more complete study could show if there are groups of patients who strongly increase the expression of ABCG2 in acute attack conditions, who maintain subclinical inflammation, with hyperuricemia for a long time. It would be important to know whether the gene responds significantly in the early stages of the disease, in the chronic state or indistinctly during acute periods, as suggested by our results. The implications of this knowledge would be reflected in more effective therapies for gout and its comorbidities.

IL-1B was the second gene significantly expressed in both cell groups in patients but not in controls (*p* < 0.05). It can indicate that neutrophils were activated by the effect of circulating sUA in patients with hyperuricemia or by MSU (in an affected joint after an acute attack). The expression of IL-1β significantly correlated in PMNLs with a history of hypertension in patients; Table 4. Hypertension has been associated with chronic inflammatory diseases, aging, diabetes, and gout [35, 36]. IL-1β is important in the inflammatory process that leads to endothelial dysfunction of blood vessels to develop hypertension. Therefore, the increase in the expression of this cytokine could be a biomarker of damage and inflammation in patients with gout and its increase together with ABCG2 in the blood could be associated with a presymptomatic state.

SLC22A12 have been associated with hyperuricemia and gout [36, 37]. In our work, we did not find an increase or decrease of SLC22A12 expression in leukocytes of patients or controls. In PBMCs from patients, we found significant positive correlations between SLC22A12 expression with metabolic syndrome, sUA and triglyceride levels (these correlations were not found in controls, data not shown). These correlations may suggest that despite the low expression of the gene, the gene could be activated by metabolic alterations in the patient. An obesity/metabolic syndrome model in mice on a high-fat diet found increased SLC22A12, GLUT9, and ABCG2 expression in the kidney after 8 weeks but not in other transporters [38], while in another study, the expression of SLC22A12 and GLUT9 was measured in mice after a diet rich in fructose, inducing metabolic syndrome and hyperuricemia in the animals, authors reported an increase in the expression of these two genes [39]. There are also different reports that link hyperuricemia with increased expression of URAT1 in the kidney, blood, and salivary gland [34, 40].

In PMNLs, as seen in Table 4, the expression of SLC22A12 correlated positively with hypertension only in patients. Associations of metabolic syndrome and obesity with variants of SLC22A12 (rs11602903 and rs11231825), which predispose patients to gout, have been reported [41, 42]. The risk of chronic kidney disease is known due to the presence of hyperuricemia, and the importance of URAT1 in different kidney diseases has recently been understood [43, 44]. An in vitro study revealed that mice previously treated with reverastrol had reduced renal SLC22A12 expression and, consequently, hyperuricemia caused by uric acid reabsorption by URAT1 in the kidney [45]. Despite the low gene expression of SLC22A12 in our study, it could be related to hyperuricemia and the consequent kidney damage in people with a longer time with the disease and poor control of hyperuricemia. In the case of patients with kidney disease, they could have overexpression of the transporter in the kidney if there are no mutations present; however, a longitudinal study is needed that considers uric acid levels, renal dysfunction, and the treatment of each patient to compare the expression of SLC22A12 in these cases [45].

In PBMCs from patients, we found correlations of ALPK1 expression with creatinine and uric acid levels. In vitro induction of the expression of ALPK1 in response to MSU has been reported in monocytes and macrophages [46], which could indicate that leukocytes express ALPK1 in response to high uric acid concentrations or subsequent local activation of monocytes and macrophages. ALPK1 is known to be associated with kidney disease through association studies, and its expression in animal models can regulate the release of the chemokines CCL2 and CCL5 in kidney cells [47]. However, we did not find a correlation with a history of kidney disease, hence, the expression of ALPK1 in PBMCs may be increased by inflammatory signals in the blood, kidney, or intestine due to frequent hyperuricemia in patients even in an asymptomatic state.

In PMNLs of patients, we found a correlation of ALPK1 with CRP, and although there were no significant differences in CRP with controls, the average CRP in patients was high (5.98 ± 4.6 mg/L); however, this increase could indicate a proinflammatory state in some patients, which together with the presence of hyperuricemia favors the recruitment of neutrophils and a new attack of gout. The correlation of ALPK1 gene expression with sUA could support this idea, whereas the correlation with serum creatinine levels could support the presence of proinflammatory factors [48]. The increased expression of ALPK1 and SLC22A12 in the blood of patients with gout could be associated with their altered metabolic status, which favors a long-term chronic condition with recurrent acute episodes if hyperuricemia and comorbidities are maintained (55). A genomic study analyzed the epistasis of ALPK1 with the loci of GLUT9, ALPK1, SLC22A12, and ABCG2, finding a positive predictive value (PPV ≥ 81%) to measure the risk of gout (OR ≥ 12.30) using variants of these genes in different populations. with gout; thus, this gene seems to continue to be linked to gout, although its importance in its progression is not evident [49].

## Conclusions

Differential gene expression of ABCG2, SLC22A12, IL-1β, and ALPK1 in peripheral blood leukocytes of primary gout patients (chronic or acute) correlated with hyperuricemia and its comorbidities, such as dyslipidemia, obesity, hypertension, metabolic syndrome, kidney disease, and high levels of creatinine in blood. The increased gene expression of the alpk1 and the urate transporters SLC22A12 and ABCG2 could be part of an altered molecular profile in response to continuous episodes of gout and comorbidities that favor inflammation. A follow-up study to analyze the variation in the gene and protein expression of these molecules will help to determine whether they can be candidates for markers of damage by the disease.

Our data suggest that the leukocytes of gout patients respond differentially to hyperuricemia and its comorbidities and increase the gene expression of ABCG2 and IL-1β compared to normouricemic and non-gout controls.

## Data Availability

All the information and results are in this manuscript for review, if required extra information upon request.

## Abbreviations

PBMCs: Peripheral blood mononuclear cells
PMNLs: Polymorphonuclear leukocytes white blood cells
sUA: Serum uric acid
CRP: C-reactive protein
BMI: Body mass index
MSU: Monosodium urate crystals
URAT1: Urate transporter 1
ATP III: Adult Treatment Panel III report
EULAR: European Alliance of associations for Rheumatology
ACR: American College of Rheumatology
GLC: Glucose
TC: Total cholesterol
TG: Triglycerides
HDL: High density lipoprotein
LDL: Low density lipoprotein
Cr: Creatinine
SE: Standard error
